# Oxygen deficient α-Fe_2_O_3_ photoelectrodes: a balance between enhanced electrical properties and trap-mediated losses†
†Electronic supplementary information (ESI) available: Full experimental details and sample characterization data can be found in the ESI. See DOI: 10.1039/c5sc00423c


**DOI:** 10.1039/c5sc00423c

**Published:** 2015-04-28

**Authors:** Mark Forster, Richard J. Potter, Yichuan Ling, Yi Yang, David R. Klug, Yat Li, Alexander J. Cowan

**Affiliations:** a University of Liverpool , Stephenson Institute for Renewable Energy , Department of Chemistry , Liverpool , L69 7ZF , United Kingdom . Email: acowan@liverpool.ac.uk; b University of Liverpool , School of Engineering , George Holt Building , Brownlow Hill , Liverpool , L69 3GH , United Kingdom . Email: rjpott@liverpool.ac.uk; c Imperial College London , Department of Chemistry , Exhibition Road , London , SW7 2AZ , United Kingdom . Email: d.klug@imperial.ac.uk; d University of California , Department of Chemistry and Biochemistry , 1156 High Street , Santa Cruz , California 95064 , United States of America . Email: yatli@ucsc.edu

## Abstract

Intrinsic doping of hematite through the inclusion of oxygen vacancies (V_O_) is being increasingly explored as a simple, low temperature route to preparing active water splitting α-Fe_2_O_3–*x*_ photoelectrodes. Whilst it is widely accepted that the introduction of V_O_ leads to improved conductivities, little else is verified regarding the actual mechanism of enhancement. Here we employ transient absorption (TA) spectroscopy to build a comprehensive kinetic model for water oxidation on α-Fe_2_O_3–*x*_. In contrast to previous suggestions, the primary effect of introducing V_O_ is to block very slow (ms) surface hole – bulk electron recombination pathways. In light of our mechanistic research we are also able to identify and address a cause of the high photocurrent onset potential, a common issue with this class of electrodes. Atomic layer deposition (ALD) of Al_2_O_3_ is found to be particularly effective with α-Fe_2_O_3–*x*_, leading to the photocurrent onset potential shifting by *ca.* 200 mV. Significantly TA measurements on these ALD passivated electrodes also provide important insights into the role of passivating layers, that are relevant to the wider development of α-Fe_2_O_3_ photoelectrodes.

## Abstract 2

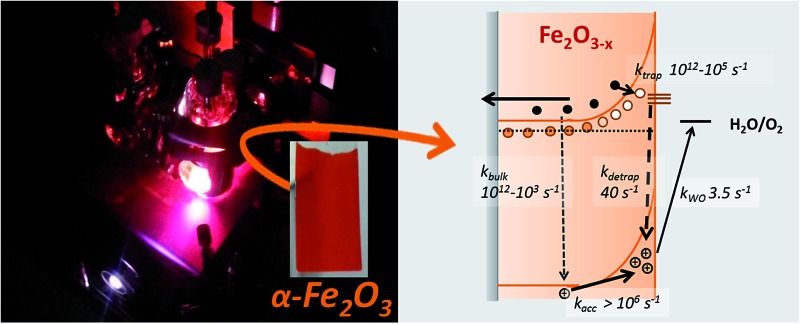
Intrinsic doping of hematite through the inclusion of oxygen vacancies (V_O_) is being increasingly explored as a simple, low temperature route to preparing active water splitting α-Fe_2_O_3–*x*_ photoelectrodes.

## Introduction

There is intense interest in the use of photoelectrochemical (PEC) systems for the production of solar fuels. Amongst the more promising photoanodes for PEC water oxidation is hematite (α-Fe_2_O_3_), a non-toxic, abundant, low-cost, and relatively inert material. The bandgap and band energies of α-Fe_2_O_3_ (∼2.1 eV) lead to a maximum theoretical solar-to-hydrogen (STH) efficiency of ∼15%,^1^ however actual achieved STH efficiencies of α-Fe_2_O_3_ photoelectrodes are substantially below this value and are typically ∼1–2%.^2^ This has been proposed to be due to multiple limiting factors, including; poor conductivity,^3^ short electron–hole lifetimes,^4^ slow oxygen evolution reaction kinetics^5^ and a low visible light absorption coefficient coupled to a short hole diffusion length (2–4 nm).^6^ Numerous approaches to improving the activity of α-Fe_2_O_3_ have been explored. Higher photocurrent densities have been achieved through nanostructuring which has the aim of increasing the concentration of charges generated close to the semiconductor liquid junction (SCLJ) to overcome the short hole diffusion length.^7^ In such nanostructured electrodes, dopants are known to be critical for photoelectrochemical activity with un-doped α-Fe_2_O_3_ electrodes being electrically insulating^7^ and often photoelectrochemically inactive.^8^ A wide variety of extrinsic dopants have now been explored including Si,^8^–^10^ Sn,^11^–^13^ Ti,^13^–^15^ Pt 16,17 and these lead to both enhanced long range charge transport and the formation of a sufficient electric field for charge separation at the SCLJ within the nanostructured domains.^8^

The introduction of oxygen vacancies (V_O_) has also recently been shown to be an effective approach to improve the activity of α-Fe_2_O_3_ photoelectrodes.^18^–^20^ A report by Li *et al.* on the decomposition of β-FeOOH nanowires in an oxygen deficient atmosphere demonstrated the highly active nature of oxygen deficient hematite (α-Fe_2_O_3–*x*_) photoelectrodes for water oxidation, with a photocurrent of 3.4 mA cm^–2^ being achieved under 100 mW cm^–2^, and in this paper we examine the factors behind the high activity of these same electrodes.^19^ The enhanced activity, ease of inclusion of V_O_, ability to process at temperatures as low as 350 °C 21 and reports of a synergistic effect of extrinsic dopants with intrinsic V_O_ sites^20^,^22^ has led to a surge of interest in the controlled inclusion of V_O_ in hematite over the last 2 years. Given the potential utility of this approach it is important that the fundamental processes associated with V_O_ inclusion are elucidated.^23^–^26^

The higher incident photon to current efficiency (IPCE) of α-Fe_2_O_3–*x*_ (64% at 1.50 V_RHE_) compared to the air annealed sample (α-Fe_2_O_3_, 0.57% at 1.50 V_RHE_) in the report of Li *et al.* correlated with the presence of Fe^2+^ sites as measured by XPS and a large increase in the measured donor density (*N*_d_).^19^ It has been known for over 25 years that the inclusions of V_O_/Fe^2+^ sites within α-Fe_2_O_3_ leads to formation of a donor band ∼80 meV below the conduction band.^27^ The inclusion of V_O_'s is suggested to improve activity through a number of mechanisms including; improved charge transport, higher charge separation yields and decreased contact resistances at the semiconductor/transparent conducting oxide interface, with various studies citing differing contributions from each effect.^13^,^21^,^23^–^26^ However, to date research has concentrated on material development and few direct measurements of the actual mechanism of enhanced activity for α-Fe_2_O_3–*x*_ are reported.

A further complication is that although large increases in photocurrent have been achieved through the inclusion of V_O_, the onset potentials remain relatively high for α-Fe_2_O_3–*x*_, typically 1.0 V_RHE_ or greater^19^,^21^ compared to as low as ∼0.6 V_RHE_ for an ALD α-Fe_2_O_3_ electrode following a high temperature (800 °C) treatment, leading to typical solar to fuels efficiencies for oxygen deficient hematite that are significantly lower than other state of the art hematites.^28^,^29^ It has been proposed by several authors that a possible cause of the high onset potentials in α-Fe_2_O_3–*x*_ is the increased concentration of surface defect states.^30^,^31^ Surface defects in hematite are known to lead to Fermi level pinning and increased levels of trap-mediated recombination, and the selective passivation of such states in stoichiometric α-Fe_2_O_3_ has proven to be a highly effective approach to improving photoelectrode activity.^30^ Surface passivation treatments on extrinsically doped photoelectrodes have included high temperature annealing steps and overlayer depositions.^29^,^32^,^33^ The hypothesised deleterious role of surface states in oxygen deficient hematite is apparently contradicted by a recent study on hydrogen treated α-Fe_2_O_3_ which reported improved linear sweep photocurrents (recorded at 50 mV s^–1^) for samples only containing Fe^2+^ defect states at the surface, compared to photoelectrodes containing both bulk and surface V_O_.^24^ It is therefore apparent that currently no consensus exists regarding the mechanism of operation of oxygen deficient hematite. In order to systematically address the high onset potential of α-Fe_2_O_3–*x*_ samples an improved understanding of the role of both bulk and surface V_O_ sites and their effects on the charge carrier kinetics is required.

Transient absorption (TA) spectroscopy and transient photocurrent (TPC) measurements offer a route to directly measure the effect of material modifications and treatments on the yield and dynamics^34^,^35^ of photogenerated charges within a PEC cell.^36^ Previous TA spectroscopic studies of α-Fe_2_O_3_ photoelectrodes have examined numerous aspects of the photophysics and chemistry of extrinsically doped α-Fe_2_O_3_, including the role of co-catalysts on hole kinetics,^31^,^32^,^37^,^38^ and the effect of bias on charge trapping and recombination, from the fs–ms timescale.^39^,^40^ Of particular significance has been the realization that a key requirement for water splitting is the ability to accumulate very long-lived holes at the SCLJ, with apparent rate constants for water splitting ranging from 0.1–6 s^–1^,^41^,^42^ correlating with a measured significant thermal barrier to hole transfer.^43^ The slow hole transfer enables recombination between bulk electrons and surface accumulated holes on the millisecond timescale in Si–Fe_2_O_3_ at <1.2 V_RHE_ which significantly lowers photoelectrochemical activity. Here we report the first TA study of a α-Fe_2_O_3–*x*_ photoelectrode in a PEC cell, with the aim of identifying the key design rules required to develop more efficient defect rich photoanodes. We have chosen to study samples prepared as originally reported by some of us^19^ as they remain amongst the most active oxygen deficient electrodes reported, with current densities reaching *ca.* 4 mA cm^–2^ under 1 sun illumination. Our detailed kinetic analysis allows to (i) elucidate the observed bias dependent activity of α-Fe_2_O_3–*x*_, yielding a complete mechanistic model of the operation of oxygen deficient electrodes and (ii) to provide critical mechanistic insights into the role of ALD Al_2_O_3_ overlayers. ALD Al_2_O_3_ is found to be particularly effective in shifting the photocurrent onset potential of α-Fe_2_O_3–*x*_ (∼0.2 V), representing a significant enhancement in photoelectrochemical water splitting efficiency.

## Results and discussion

**Fig. 1:**
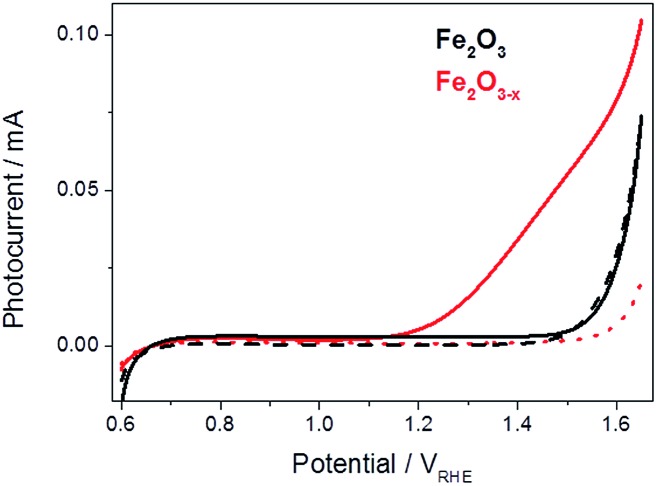
Linear sweep voltammograms for α-Fe_2_O_3_ (black) and α-Fe_2_O_3–*x*_ (red), 5 mV s^–1^, under low power white light illumination (solid line, *ca.* 0.1 sun) and in the dark (dashed line).

For this study fresh α-Fe_2_O_3_ (air annealed) and α-Fe_2_O_3–*x*_ (oxygen deficient) films were prepared as previously described, details can be found in the ESI.† 19 The photoelectrochemical activities of both α-Fe_2_O_3_ and α-Fe_2_O_3–*x*_ were assessed through linear sweep voltammograms measured in 1 M NaOH using a portion of the output of a 75 W Xe lamp (*ca.* 0.1 sun) scanning at 5 mV s^–1^. We employ this low power light source in our mechanistic study as it allows us to measure the photoelectrochemical response of the sample inside the transient spectrometer using the same cell as in the TA measurements. In line with previous reports we observe a strong photocurrent from α-Fe_2_O_3–*x*_ assigned to photoelectrochemical water oxidation, Fig. 1.^41^,^44^ It is known that IPCE values for these α-Fe_2_O_3–*x*_ films exceed 60% at 1.5 V_RHE_, leading to photocurrents of *ca.* 4 mA cm^–2^ under 100 mW cm^–2^, however a key concern remains the high onset potential,^19^ observed here at *ca.* 1.15 V_RHE_, Fig. 1. In contrast to α-Fe_2_O_3–*x*_, the air annealed (550 °C) α-Fe_2_O_3_ sample shows no significant photocurrent (0.6–1.5 V_RHE_). The lack of activity of this control α-Fe_2_O_3_ sample, chosen due to its similar morphology, preparation route and light harvesting properties, indicates that the presence of V_O_ is critical for enabling photoelectrochemical water splitting in these otherwise un-doped films.

**Fig. 2:**
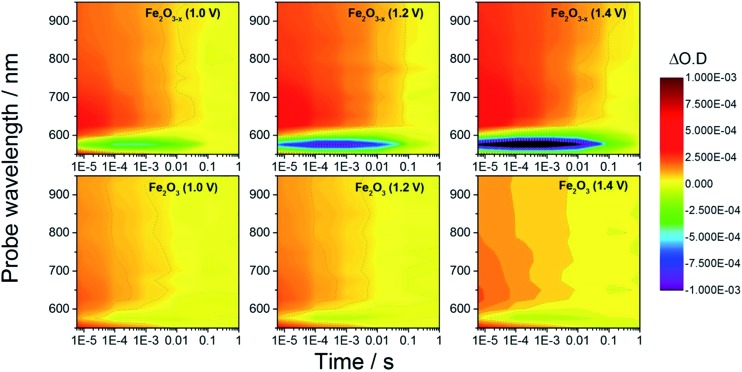
TA spectra of α-Fe_2_O_3_ and α-Fe_2_O_3–*x*_ at various applied potentials (*vs.* RHE) in 1 M NaOH with the photoanodes excited from the electrolyte/electrode side (EE) with a 355 nm (6 ns pulse) laser.

In order to rationalize the observed photoelectrochemical response of the hematite samples we have measured TA spectra following UV excitation (355 nm, 6 ns, 100 μJ cm^–2^) at a range of applied biases, Fig. 2. The excitation energy employed leads to photo-generated carrier densities several orders of magnitude lower than the calculated oxygen vacancy density (ESI, S7†). A broad positive transient absorption is measured at wavelengths greater than 600 nm in all of the spectra. In line with numerous past TA studies on Si-doped and Nb-doped α-Fe_2_O_3_ photoelectrodes,^40^,^43^,^45^ the TA feature at *λ* > 600 nm is assigned to photoholes in α-Fe_2_O_3_ (see Fig. S4† for a schematic explanation) and this assignment is further affirmed in TA experiments employing a hole scavenger (H_2_O_2_, *vide infra*) which show a decreased signal at *λ* > 600 nm. The bias dependent TA spectra also contain a bleach (decrease in optical density) centred at *ca.* 580 nm which is seen to increase in magnitude with applied bias. It has been proposed that the feature at this wavelength is due to localized states close to the band edge, possibly related to the presence of oxygen vacancies.^27^,^40^,^46^ Under positive bias the trap state occupancy is lowered, enabling the promotion of a valence band electron to the vacant trap state upon absorption of a visible photon (580 nm). Using both fs and μs TA measurements, Pendlebury *et al.* have shown that following UV excitation of α-Fe_2_O_3_ held at potentials significantly positive of the flat band potential, rapid photoelectron trapping can occur leading to a bleaching of this 580 nm feature, (see Fig. S4†).^39^,^40^ We also assign the bleach at ∼580 nm to photoelectron trapping at localized states which is primarily occurring on the sub-microsecond timescale.

**Fig. 3:**
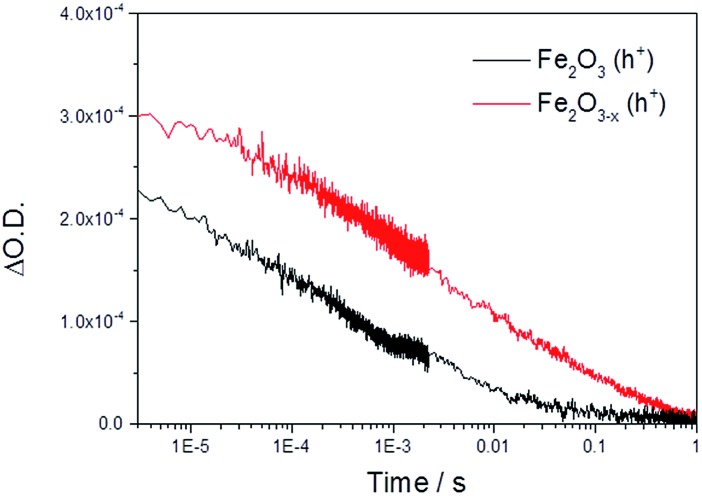
TA decay traces at 700 nm of photoholes on α-Fe_2_O_3_ and α-Fe_2_O_3–*x*_ at 1.4 V_RHE_ in 1 M NaOH following UV excitation (355 nm).

Initially we concentrate our study on the yield and kinetics of photoholes in α-Fe_2_O_3_ and α-Fe_2_O_3–*x*_. At all potentials investigated we note an increased yield of photoholes in Fe_2_O_3–*x*_ at the earliest timescales studied (2 μs), Fig. 2. The increased photohole yield at 2 μs may indicate more efficient initial charge separation in the oxygen deficient hematite, however a detailed study of the photohole kinetics at a single potential (1.4 V_RHE_) shows only a ∼35% difference in photohole yield at 2 μs (assuming a similar extinction coefficient for both materials), Fig. 3. This suggests that a previously proposed enhancement^19^,^38^ in initial charge separation yield in V_O_ rich materials with higher electron densities is not likely to be a significant factor in rationalizing the differences in activity of α-Fe_2_O_3_ and α-Fe_2_O_3–*x*_. Instead, of greater significance is the rate of photohole decay in α-Fe_2_O_3_ (*t*_50%_ = 0.27 ms) and α-Fe_2_O_3–*x*_ (*t*_50%_ = 1.20 ms) at 1.4 V_RHE_.^47^ Previous studies have indicated that the improved conductivity of α-Fe_2_O_3–*x*_ would be expected to aid both initial charge separation and electron transport/extraction which reduce bulk electron–hole recombination and thereby increase the photohole lifetime.^21^,^48^

**Fig. 4:**
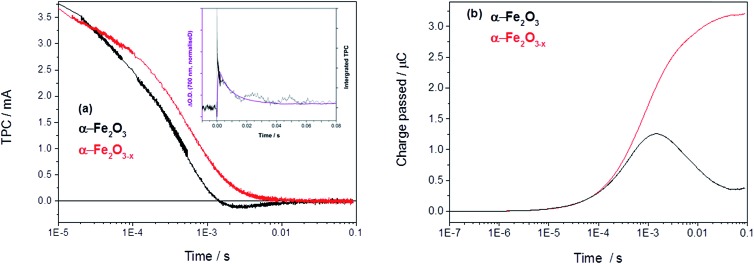
(a) TPC decays of α-Fe_2_O_3_ and α-Fe_2_O_3–*x*_ held at 1.4 V_RHE_ with an inset showing the overlay of the decay of the TA with the integrated TPC in Fe_2_O_3_ at 1.4 V_RHE_ (Fig. S6†) and (b) the converted total charge passed following UV excitation (355 nm, 6 ns).

In order to asses if such a change in photoelectron dynamics is the cause of the improved activity of α-Fe_2_O_3–*x*_ we have also measured the transient photocurrent (TPC) between the hematite working electrodes and the counter electrode following UV excitation. At 1.4 V_RHE_ the TPC traces of both α-Fe_2_O_3_ and α-Fe_2_O_3–*x*_ are relatively similar at early times (<0.5 ms) indicating that, contrary to expectations, initial photoelectron extraction to the external circuit can occur effectively even in the un-doped α-Fe_2_O_3_ sample.^19^ Instead we find that the difference in photohole kinetics and photoelectrochemical activity is due to a slow back electron transfer from the external circuit into the air annealed α-Fe_2_O_3_, which we see as a negative current on the timescale of 1–10 ms after photon absorption, Fig. 4. For α-Fe_2_O_3_ samples the total charge re-injected from the external circuit approximately matches that initially extracted from the α-Fe_2_O_3_ photoelectrode at all potentials studied (0.8–1.4 V), leading to minimal net photocurrent in our transient study, in line with the steady state photocurrent measurements, Fig. 4 and S5.†

An overlay of the kinetics of charge reinjection (TPC) and hole decay (TA) on α-Fe_2_O_3_ (*τ* = 10 ms at 1.4 V_RHE_, Fig. 4 inset and Fig. S6†) shows an excellent agreement indicating that bulk electrons are recombining with the accumulated photoholes measured in the TA experiment, leading to reinjection of electrons from the external circuit. Experiments in the presence of H_2_O_2_ in the following section allow us to assign this recombination to be occurring with surface trapped holes. This is in agreement with a very recent study on Si–Fe_2_O_3_ where TPC measurements showed the presence of a recombination process between surface trapped holes and bulk α-Fe_2_O_3_ electrons on the milliseconds timescale, that led to a flow of electrons back into the photoelectrode from the external circuit.^41^ In contrast, with α-Fe_2_O_3–*x*_ electrodes we do not observe any slow re-injection of electrons into the film, Fig. S5.†

The lack of back electron transfer into α-Fe_2_O_3–*x*_ electrodes can be rationalized by Mott–Schottky measurements of the two films (Fig. S7†) that show a higher donor density (*N*_d_) in the V_O_ rich photoelectrode (α-Fe_2_O_3–*x*_*N*_d_ = 1.2 × 10^20^ cm^–3^, α-Fe_2_O_3_*N*_d_ = 6.7 × 10^19^ cm^–3^) with flat-band potentials of 0.4 V_RHE_ (α-Fe_2_O_3–*x*_) and 0.34 V_RHE_ (α-Fe_2_O_3_).^19^ Whilst absolute values of donor densities for a nanostructured film obtained through a Mott–Schottky analysis should be treated with caution, the relative change between these two samples with similar morphologies (Fig. S3†) is significant. This two-fold increase in *N*_d_ in the α-Fe_2_O_3–*x*_ electrode significantly decreases the width of the space charge layer (*W*_sc_), Fig. S7† for illustrative calculations. Our TAS studies confirm that greater localised band bending in the surface region of α-Fe_2_O_3–*x*_ is a significant factor in rationalising the enhanced activity of α-Fe_2_O_3–*x*_, as hypothesised in several previous studies.^19^,^21^,^30^,^31^,^38^ However it is not found to be due to more efficient initial charge separation or injection kinetics but instead the blocking of the back flow of electrons from the bulk towards the SCLJ preventing recombination with surface accumulated photoholes.

A simple model of the kinetic competition between the bias dependent back electron–hole recombination pathway and the rate of water oxidation has been shown elsewhere to account for observed photoelectrochemical activity of Si–Fe_2_O_3_ electrodes without the need for the inclusion of inter-band trap states.^41^ Specifically the photocurrent onset potential of a Si-doped α-Fe_2_O_3_ photoelectrode, with a similar *N*_d_ (∼10^20^ cm^3^) to the α-Fe_2_O_3–*x*_ samples examined here (1.2 × 10^20^ cm^–3^), correlated with the potential at which back electron–hole recombination became slow enough to enable surface hole accumulation and water oxidation to occur (*ca.* 1.0 V_RHE_). Here we find that back electron–hole recombination in α-Fe_2_O_3–*x*_ is blocked at potentials as low as 0.8 V_RHE_ (Fig. S5†) and no correlation is noted with the photocurrent onset potential, *ca.* 1.15 V_RHE_ (Fig. 1). This gives rise to an intriguing question, given that our TPC and TA measurements show that holes can be accumulated at potentials as low as 0.8 V_RHE_ and that the slow back electron–hole recombination pathway has been prevented, why is the photocurrent onset potential for water oxidation so positive (1.15 V_RHE_) with α-Fe_2_O_3–*x*_ photoelectrodes?

### Site of photohole accumulation in α-Fe_2_O_3–*x*_

**Fig. 5:**
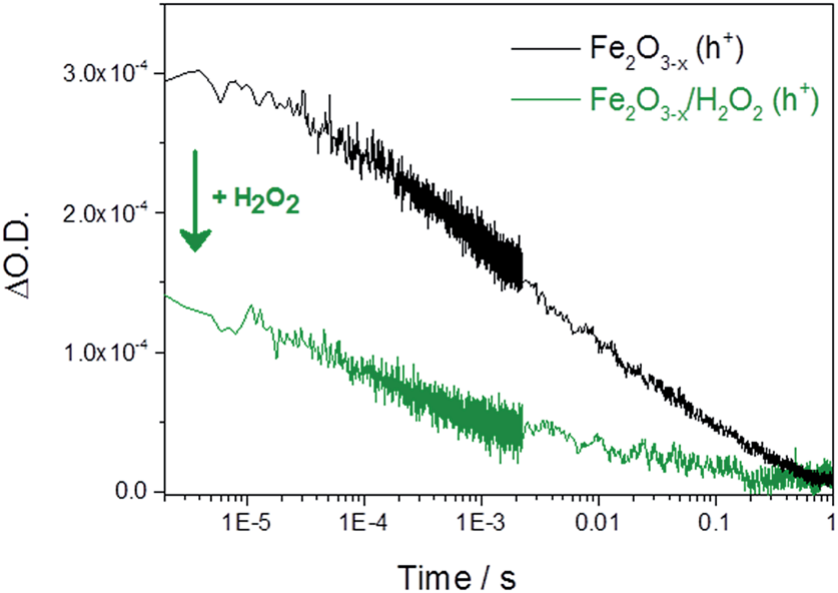
TA decay traces recorded at 700 nm of photoholes on α-Fe_2_O_3–*x*_ at 1.4 V_RHE_ in 1 M NaOH in the absence and presence of a hole scavenger (0.5 M H_2_O_2_).

It is therefore important to identify if the photoholes measured in α-Fe_2_O_3–*x*_ using TA spectroscopy on the μs–ms timescale are accumulating at or close to the SCLJ, as hole trapping elsewhere in this defect rich material may account for the high photocurrent onset potential. In order to distinguish these two cases we explore the response of the hole dynamics to the presence of a hole scavenger. Hydrogen peroxide is a commonly used hole scavenger due to its near unity efficiency for the removal of holes in α-Fe_2_O_3_ that are at or close to the SCLJ.^49^ In the presence of 0.5 M H_2_O_2_ we observe a photocurrent at potentials as low as 0.7 V_RHE_ on both α-Fe_2_O_3_ and α-Fe_2_O_3–*x*_ confirming that (i) initial charge separation is effective in both materials at potentials well below the water oxidation photocurrent onset potential and (ii) that in both samples the photoholes are able to reach the surface to participate in oxidation reactions, Fig. S8.† TA experiments in the presence of H_2_O_2_ show a decrease in the hole yield at the earliest time-scales studied (2 μs) in Fe_2_O_3–*x*_ when compared to experiments in the absence of a hole scavenger. This indicates that holes are present at the SCLJ and are transferring into solution at time scales earlier than studied here, providing a lower limit for the lifetime of transport and accumulation of holes at the SCLJ (∼2 μs), Fig. 5.

### Role of trap state mediated recombination

**Fig. 6:**
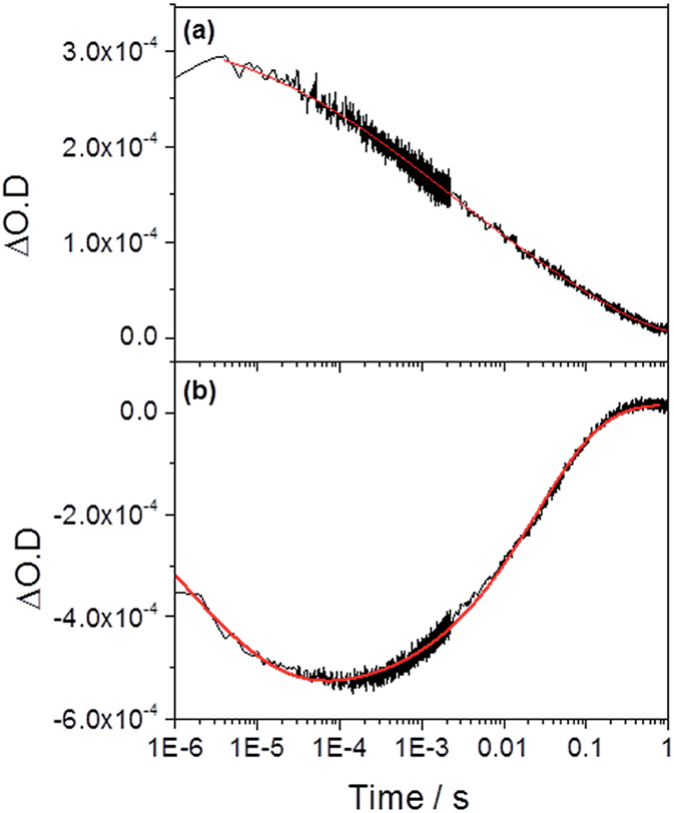
TA traces recorded at (a) 700 nm (photoholes) and (b) 580 nm (electron trap state) in α-Fe_2_O_3–*x*_ at 1.4 V_RHE_ following UV excitation (355 nm) in 1 M NaOH. The red lines correspond to the fitted functions identified in the main text. Full parameters can be found in the ESI.†

We now turn to the potential role of electron trap states in rationalizing the behaviour of α-Fe_2_O_3–*x*_ as it has been proposed that oxygen vacancies may act as a “mixed blessing”^38^ with the enhanced electrical properties being balanced with a potential increase in trap-mediated electron–hole recombination at the defect sites introduced.^30^ Following absorption of a UV photon we initially observe a bleaching at 580 nm for α-Fe_2_O_3–*x*_ which is assigned to photoelectron trapping at this inter-band state, Fig. 6(b).^39^ The slow recovery on the millisecond timescale of the trap state feature is due to the subsequent de-trapping of the photoelectrons, as shown by the dynamics in Fig. 6(b). In line with this assignment, the change in occupancy of the 580 nm trap state at 1.4 V_RHE_ is well fitted to the integrated rate equation for an intermediate species in a consecutive reaction scheme (A → B → C) (Fig. 6(b)), eqn (1).
1

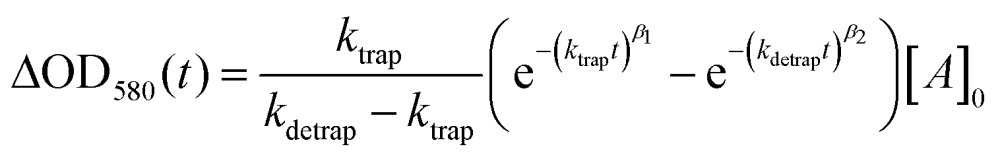

where the rate constants *k*_trap_ and *k*_detrap_ correspond to the rate of photoelectron trapping and de-trapping (*ca.* 7 × 10^5^ s^–1^ and 40 s^–1^ respectively), *β* a stretching exponent and [*A*]_0_ a pre-exponential factor related to the initial yield of photoelectrons. Photoelectron trapping has been shown to occur on the ps–μs timescales and it is likely we have only fitted the tail of the trapping process here.^39^

**Fig. 7:**
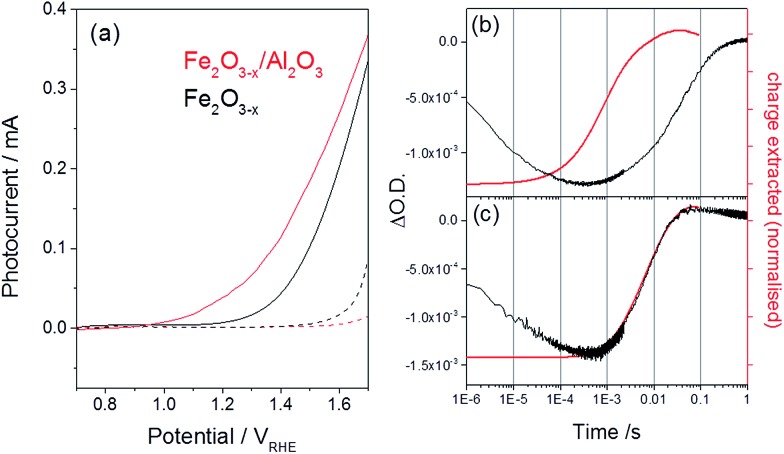
(a) Photocurrent of α-Fe_2_O_3–*x*_ before (black) and after deposition of an ALD Al_2_O_3_ layer (1 nm, red trace) recorded using a low power 75 W Xe lamp, dark traces are shown as dashed lines. (b) Overlay of the TA trace recorded at 580 nm, assigned to an interband trap state, and the normalized charge extracted for (b) α-Fe_2_O_3–*x*_ and (c) α-Fe_2_O_3–*x*_/Al_2_O_3_ following UV excitation (355 nm) at 1.4 V_RHE_. All traces are recorded in 1 M NaOH.^51^

In previous studies the de-trapping rate of photoelectrons in extrinsically doped hematite correlated to the TPC decay rate as well as a distinct fast decay component in the hole population, leading to an assignment of both electron extraction and electron–hole recombination following de-trapping in the bulk of the electrode.^40^ In contrast, on α-Fe_2_O_3–*x*_ a sample with a high concentration of surface defect sites^21^,^50^ it is proposed that a significant level of electron trapping occurs at the surface, closer to the site of photohole accumulation leading to higher electron–hole recombination losses upon detrapping. We are able to assign electron–hole recombination as the fate of the de-trapped electrons due to the lack of correlation between the TPC decay rate and the rate of recovery of the 580 nm signal (Fig. 4, 6 and 7(b)), which indicates that the electrons are unable to reach the external circuit. In the absence of photoelectron extraction it is expected that electron–hole recombination will occur. We can also conclude that a significant level of recombination with trap state electrons occurs close to the SCLJ as in the previous section TA experiments demonstrated that the a large portion of the photohole population reached the surface of α-Fe_2_O_3–*x*_ within the time resolution of our experiment (2 μs), *i.e.* prior to photoelectron detrapping.

**Scheme 1:**
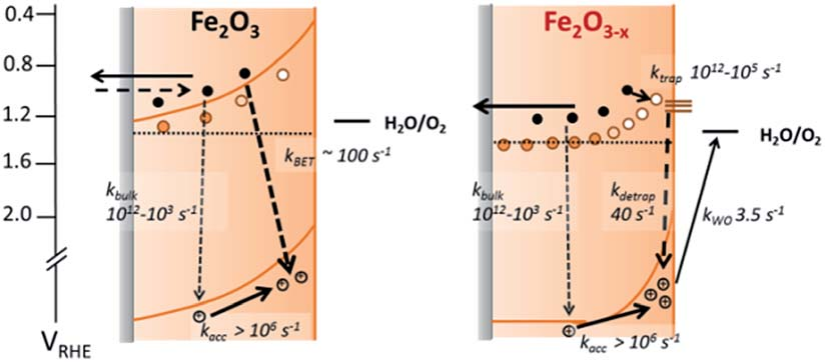
Simplified energy diagrams and processes involving photogenerated charges following UV laser excitation of α-Fe_2_O_3_ and α-Fe_2_O_3–*x*_ under a positive bias. Rate constants are those determined at 1.4 V_RHE_ as described in the main text. In contrast to α-Fe_2_O_3_ which has been air annealed, no slow recombination between surface accumulated holes and bulk electrons (*k*_BET_) is observed for α-Fe_2_O_3–*x*_.

We further confirm the presence of a significant level of trap-mediated recombination in the oxygen deficient hematite through a detailed kinetic analysis of the photoholes. In the absence of a recombination process between surface trapped holes and bulk α-Fe_2_O_3_ electrons the photohole TA kinetics of α-Fe_2_O_3–*x*_ at 1.4 V_RHE_ would consist of three primary kinetic processes on the μs–s timescale corresponding to fast (μs) electron–hole recombination in the bulk, surface electron–hole recombination and hole transfer into solution. An excellent fit of the TA signal of α-Fe_2_O_3–*x*_ at 700 nm can indeed be achieved using a triphasic stretched exponential function, with the rate of bulk electron–hole recombination corresponding to the decay of the integrated TPC signal at the same potential (*k*_bulk_ ∼ 2 × 10^3^ s^–1^), the surface recombination rate constant matching the rate constant for photoelectron de-trapping (*k*_detrap_ ∼ 40 s^–1^), confirming the occurrence of trap-mediated recombination at the SCLJ, and a hole transfer rate constant into solution of *k*_WO_ ∼ 3.5 s^–1^ (full fitting parameters are in the ESI†), Fig. 6(a). The assignment of the slowest kinetic phase of the hole decay is in line with previously measured rate constants for water oxidation on α-Fe_2_O_3_ 40 which have ranged from ∼0.2–6 s^–1^ 42 and is further supported by a plot of the yield of very long-lived photoholes (at 200 ms) *versus* the applied bias which strongly correlates with the measured photocurrent response of α-Fe_2_O_3–*x*_, (Fig. S9†). Our analysis is also further supported by fitting of the hole trace in Fig. 5, which is well fitted with a biphasic stretched exponential function, with *k*_bulk_ and *k*_detrap_ present, but *k*_WO_ absent, confirming the nature of this slowest kinetic component, (ESI Fig. S12†). We are able to now construct a full kinetic scheme for the key steps for water oxidation on α-Fe_2_O_3–*x*_, (Scheme 1). Interestingly the amplitude of the fitting components, *i.e.* the separate populations of holes that are decaying by each pathway, is approximately the same for trap-mediated recombination and hole transfer into solution even at 1.4 V_RHE_ demonstrating that a high level of trap mediated recombination is a critical factor limiting the efficiency of oxygen deficient hematite.

In light of our TA studies that highlight the role of α-Fe_2_O_3–*x*_ surface states in mediating recombination, and show that the presence of oxygen vacancies also leads to a higher donor concentration and a narrower depletion layer suppressing the back electron injection, we are able to propose a route to both improve the activity of oxygen deficient hematite's and to test the mechanistic model proposed in Scheme 1. It is known that the preparation of α-Fe_2_O_3–*x*_, by thermal decomposition of β-FeOOH in an oxygen deficient atmosphere,^19^ leads to the formation of V_O_ both throughout α-Fe_2_O_3–*x*_ and at the surface.^24^ Removal of the states solely on the Fe_2_O_3–*x*_ surface by passivation, whilst maintaining a suitably high concentration of V_O_ both within the bulk and close to the SCLJ may be anticipated to be a route to obtaining both the desired improved *N*_d_ whilst lowering the level of trap-mediated recombination. Previously, passivation of surface states of α-Fe_2_O_3_ has been achieved through the use of high temperature annealing steps,^29^ the deposition of catalytic species including CoPi, IrO_*x*_, NiFeO_*x*_ 32,37 and the use of inert metal oxides such as Al_2_O_3_ and Ga_2_O_3_ overlayers grown by atomic layer deposition (ALD).^31^,^33^,^38^

The deposition of thin Al_2_O_3_ layers appear particularly promising as the low temperatures (120 °C) required and the reduced oxygen pressures for ALD deposition are expected to limit the loss of bulk oxygen vacancies in α-Fe_2_O_3–*x*_. ALD Al_2_O_3_ layers have been shown to decrease the photocurrent onset potentials of Si-doped α-Fe_2_O_3_ electrodes by ∼100 mV,^9^ with a decrease in the surface capacitance of the Al_2_O_3_ coated α-Fe_2_O_3_ electrodes also reported,^52^,^53^ indicating the passivation of surface trap states. Here we use ALD to form a 1 nm Al_2_O_3_ layer. This thickness is chosen as previous experiments with Si–Fe_2_O_3_ 9 have indicated that it provides reasonable coverage and stability for the duration of an experiment whilst remaining thin enough to allow for hole tunnelling transfer from the Fe_2_O_3_ to water. We find that the addition of an ALD layer on α-Fe_2_O_3–*x*_ leads to a large cathodic shift in the photocurrent onset potential (∼200 mV, 0.95 V_RHE_) and an overall increase in the magnitude of the photocurrent, Fig. 7(a) (150 W Xe lamp). Al_2_O_3_ was also deposited onto an air annealed α-Fe_2_O_3_ sample (Fig. S10†) however photocurrent measurements showed no improvement in activity confirming that sub-surface oxygen vacancies are required for photoelectrochemical activity in otherwise un-doped samples.

To confirm that the improved photoelectrochemical activity of the α-Fe_2_O_3–*x*_/Al_2_O_3_ sample is related to the hypothesised decrease in trap mediated surface electron–hole recombination, we have also measured the TA kinetics of the 580 nm trap states at 1.4 V_RHE_ following surface treatment, Fig. 7(b). To the best of our knowledge this report represents the first TA study of the role of an ALD Al_2_O_3_ passivation treatment on hematite. A large bleaching of the TA signal is observed at 580 nm even after the Al_2_O_3_ treatment showing that a significant concentration of trap states remains within the bulk of the α-Fe_2_O_3–*x*_/Al_2_O_3_ sample. Critically we also note a near four-fold increase in the rate of de-trapping with the α-Fe_2_O_3–*x*_/Al_2_O_3_ sample (*k*_detrap_ ∼ 150 s^–1^) indicating that photoelectrons in the ALD treated sample are trapped at energetically shallower sites. In contrast to the untreated α-Fe_2_O_3–*x*_ sample, where de-trapping leads to significant levels of recombination with surface trapped holes, we now see an excellent agreement between the recovery of the TA trap state band at 580 nm and the rate of charge extraction as measured by TPC for α-Fe_2_O_3–*x*_/Al_2_O_3_, Fig. 7(b and c), further supported by the observation of an increase in the yield of very long-lived photoholes in the presence of the Al_2_O_3_ overlayer (Fig. S11†). This leads to the conclusion that detrapped electrons are reaching the external circuit in appreciable quantities. We assign the change in kinetics of the transient photocurrent and trap state occupancy to the passivation of solely the surface trap states. In the absence of a significant concentration of surface trap states, photoelectrons are able to be trapped at interband trap states in the α-Fe_2_O_3–*x*_ bulk, which are spatially separated from the population of surface accumulated holes, limiting trap-mediated recombination and enabling electron transport to the external circuit, thus lowering the photocurrent onset potential.

## Conclusions

The annealing of hematite in oxygen deficient atmospheres is being increasingly explored as an approach to improving the electrical properties of the photoelectrode, however conflicting reports exist regarding the mechanism of enhancement.^40^,^52^ In contrast to previously proposed mechanisms which have often indicated improved charge transport as being a significant factor,^18^ we find that the primary effect of the introduction of oxygen vacancies is to block the slow recombination of bulk electrons with surface accumulated holes, the so called “back electron recombination” pathway at even moderate applied biases (0.8 V_RHE_).

A key target of mechanistic research is to provide design rules for rational material development and here we achieve that goal, by both proposing and verifying a modification to address the very anodic photocurrent onset potential of α-Fe_2_O_3–*x*_. We have investigated the effect of an ALD surface passivation treatment on both the photoelectrochemistry of α-Fe_2_O_3–*x*_ leading to validation of our mechanistic model and an improvement in the photocurrent onset potential of 0.2 V. Furthermore we report, to the best of our knowledge, the first TA measurements on ALD Al_2_O_3_ coated hematite providing important insights into this widely used surface treatment.

## Supplementary Material

Supplementary information
