# Supplementary material for: Oxygen deficient α-Fe2O3 photoelectrodes: a balance between enhanced electrical properties and trap-mediated losses
Source: Chem Sci. 2015 Apr 28;6(7):4009–16. doi: 10.1039/c5sc00423c (PMC5497273; doi:10.1039/c5sc00423c)
Supplement: Supplementary file 1 [file SC-006-C5SC00423C-s001.pdf]

Electronic Supplementary Material (ESI) for Chemical Science.
This journal is © The Royal Society of Chemistry 2015

Oxygen Deficient α-Fe_2O_3 Photoelectrodes: A Balance Between Enhanced Electrical Properties and Trap-Mediated Losses

Mark Forster,[a] Richard J. Potter,[b] Yichuan Ling,[d] Yi Yang, David R. Klug,[c] Yat Li[d] and Alexander J. Cowan[a]*

Electronic supplementary information

Contents:

1. Experimental

1.1 Materials

1.2 Apparatus

2. Characterisation and supporting data

2.1 UV-Vis

2.2 XRD

2.3 SEM

2.4 Scheme of TA assignments at λ > 600 nm and 580 nm

2.5 Steady state photocurrents measurements at various applied potentials

2.6 Overlay of the decay of the TA with the integrated TPC in Fe_2O_3 at 1.4 V_{RHE}

2.7 Mott-Schottky and space charge layer width calculations

2.8 Linear sweep voltammograms inn the presence of H_2O_2 hole scavenger

2.9 Fitting parameters for 700 nm TA signal

2.10 Plot of photocurrent vs. 700 nm hole intensity

2.11 Photocurrents for Al_2O_3 coated Fe_2O_3

2.12 Photohole (700 nm) intensities before and after Al_2O_3 coating

2.13 Fitting of TA trace recorded at (a) 700 nm (photoholes) in α-Fe2O3-x in the presence of hydrogen peroxide hole scavenger

1. Experimental

Synthesis of α-Fe_2O_3 and α-Fe_2O_{3-x}

Briefly, β-FeOOH nanowire films were first prepared on a FTO substrate through hydrolysis of $FeCl_3$ (0.15 M) in a high ionic strength (1 M $NaNO_3$), and low pH value (pH 1.5, adjusted by HCl) environment at 95°C for 4 hr. β-FeOOH films were then sintered in air at 550°C for 2 hr to form α-Fe_2O_3. For preparing α-Fe_2O_{3-x}, β-FeOOH films were annealed in a tube furnace in an oxygen-deficient atmosphere (N_2 + air). The tube furnace was vacuumed down to 15 Torr, and then refilled with ultrahigh purity N_2. The sintering of β-FeOOH was carried out at 550°C for 2 hours at 740 Torr with a continuous N_2 flow (50 sccm), XRD confirmed that the β-FeOOH nanowires were completely converted into hematite for both the α-Fe_2O_3 and α-Fe_2O_{3-x} samples (figure S2), in line with the past study. The morphology of the films have also been examined by SEM (figure S3) and have been found to be similar for both the α-Fe_2O_{3-x} and α-Fe_2O_3 samples, similar to those previously reported (*ca.* 70 nm by 700 nm).[19] Mott-Schottky analysis confirms the expected higher donor density in the oxygen deficient film (figure S7). Samples were found to be suitably stable for regular use in TA measurements over a 3 month period with no noticeable detrition in activity.

Al_2O_3 ALD deposition

Al_2O_3 was deposited by thermal ALD using trimethyl-aluminium (TMA) and water in an Oxford Instruments OpAL reactor. The reactor was modified by the addition of a process controlled 'hold' valve between the pump and the process chamber, enabling precursor 'soak' steps. Soak steps were used in the process to ensure good film density and to promote conformal coating of the highly textured hematite surface. Coating was carried out at a low process temperature of 120°C, selected to avoid accidental modification of the hematite samples. Films were deposited using 10 cycles of ALD targeting ~1 nm of Al2O3, using the following sequence:

{(50 ms TMA Dose)(10 s TMA hold)(10 s purge)(30 ms H_2O)(10 s H_2O hold)(10 s purge)}

The growth rate of the Al_2O_3 was estimated using a Rudulph Auto EL IV ellipsometer operating at 633 nm using silicon 'witness' samples. This confirmed that 10 cycles gave close to 1 nm of Al_2O_3. In line with previous reports,[1] the effect of Al_2O_3 layer is found to be retained for at least 20 minutes in 1 M NaOH indicating a reasonable level of stability and we are able to obtain kinetic data at up to two wavelengths.

Electrochemical measurements

A custom designed H-photoelectrochemical cell containing an α-Fe_2O_3 photoanode, illuminated (*ca.* 0.5 cm^{-2} illuminated area) from the electrolyte-electrode (EE) side, a platinum gauze counter electrode and a 3 M KCl Ag/AgCl reference electrode (SSE, Bioanalytical Systems Ltd.) protected from the electrolyte solution by a KCl double junction was used in all experiments. Potentials quoted in the text are converted to RHE using equation 1.

$$E_{RHE} = E_{Ag/AgCl} + 0.059pH + E^0_{Ag/AgCl} \qquad [1]$$

Where $E^0_{Ag/AgCl}$ = 0.205 V at room temperature. The electrolyte used throughout was 1 M NaOH (Aldrich, pH 13.6) prepared with Milli-Q water (Millipore Corp, 18.2 MΩ cm at 25°C) and prior to all experiments it was thoroughly degassed with a stream of argon for at least 20 minutes. Hole scavenging experiments were carried out in the presence of 0.5 M H_2O_2 in the electrolyte. Due to the low stability of Al_2O_3 at high pH values the electrolyte was purged thoroughly prior to addition to the electrochemical cell which was held under an argon atmosphere. Linear sweep voltammograms were recorded both in the dark and under illumination with a 75 W Xe lamp (*ca.*10 mW cm^{-2}) using either a PalmSens[3] (PalmSensBV) or a Ministat (Thomson Scientific) potentiostat typically at 5 mV s^{-1} unless otherwise stated.

Transient measurements

The TA apparatus has been described elsewhere.[2] Briefly, the third harmonic of a Nd:YAG laser (Continuum, Surelite I-10, 355 nm, 4-6 ns pulse width) operating at 0.33 Hz is the UV excitation source. The repetition rate of the laser was chosen to ensure that all photogenerated charge carriers had fully decayed prior to the next excitation event. A laser intensity of *ca.*200 µJ cm^{-2} at 355 nm was incident on the photoelectrochemical cell, leading to an intensity of *ca.*100 µJ cm^{-2} at the sample. We note that high pump laser intensities can lead to excessively high electron-hole recombination rates and in mind of this we use the relatively low excitation power of *ca.*100 µJ cm^{-2} throughout, in order to limit/eliminate this effect. There are a number of past studies on hematite regarding these laser intensities which give us confidence that the parameters used here are relevant in providing useful information.[3] A 75 W Xe lamp (OBB Corp.) coupled to monochromator (OBB Corp., set to 4 nm resolution) acts as the probe light and the change in optical density of the sample is calculated by measuring the transmitted light using a Si Photodiode (Hamamatsu) and a homemade amplification system coupled to both an oscilloscope (Textronix TDS 220) and data acquisition card (National Instruments NI-6221). In order to generate the TA spectra at a range of biases, 200 laser shots per wavelength were recorded. Accurate kinetic traces were recorded in a separate experiment using a minimum of 500 shots per wavelength. All TA experiments were carried out on the PEC cell used for electrochemical measurements, under potentiostatic control at the potential indicated in the text. Transient photocurrent measurements were recorded using the same apparatus with the light from the Xe probe lamp blocked. The current was obtained by measuring the voltage drop across a 47 ohm resistor in series with the working and counter electrode.

2. Supporting data

2.1 UV-Vis

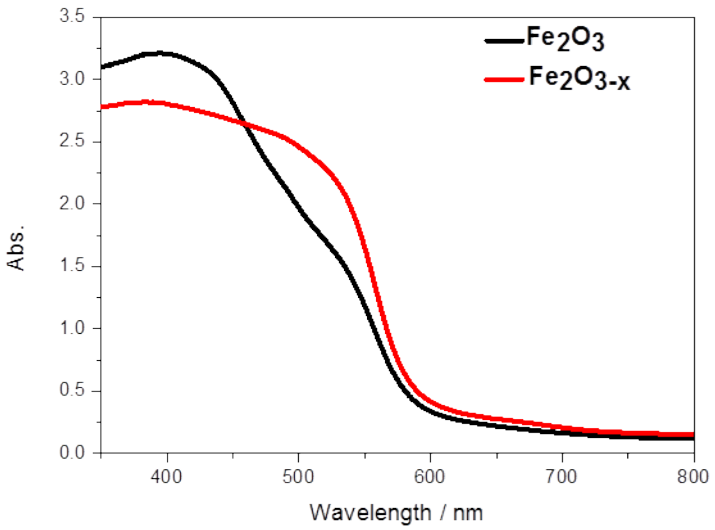

Figure S1. UV-Vis spectra of Fe_2O_3 and Fe_2O_{3-x}

2.2 XRD

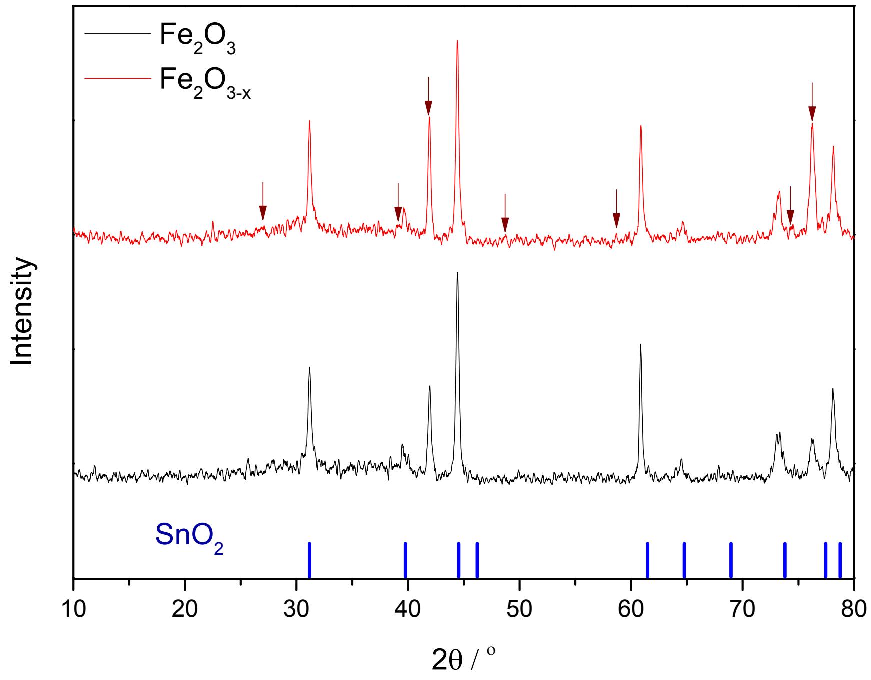

Figure S2. XRD of Fe_2O_3 and Fe_2O_{3-x} collected on FTO substrate using a Panalytical X'Pert PRO HTS X-Ray Diffractometer. Diffraction patterns were recorded from 10 to 80° 2 theta with a step size of 0.017° at 1.6° per minute.

2.3 SEM

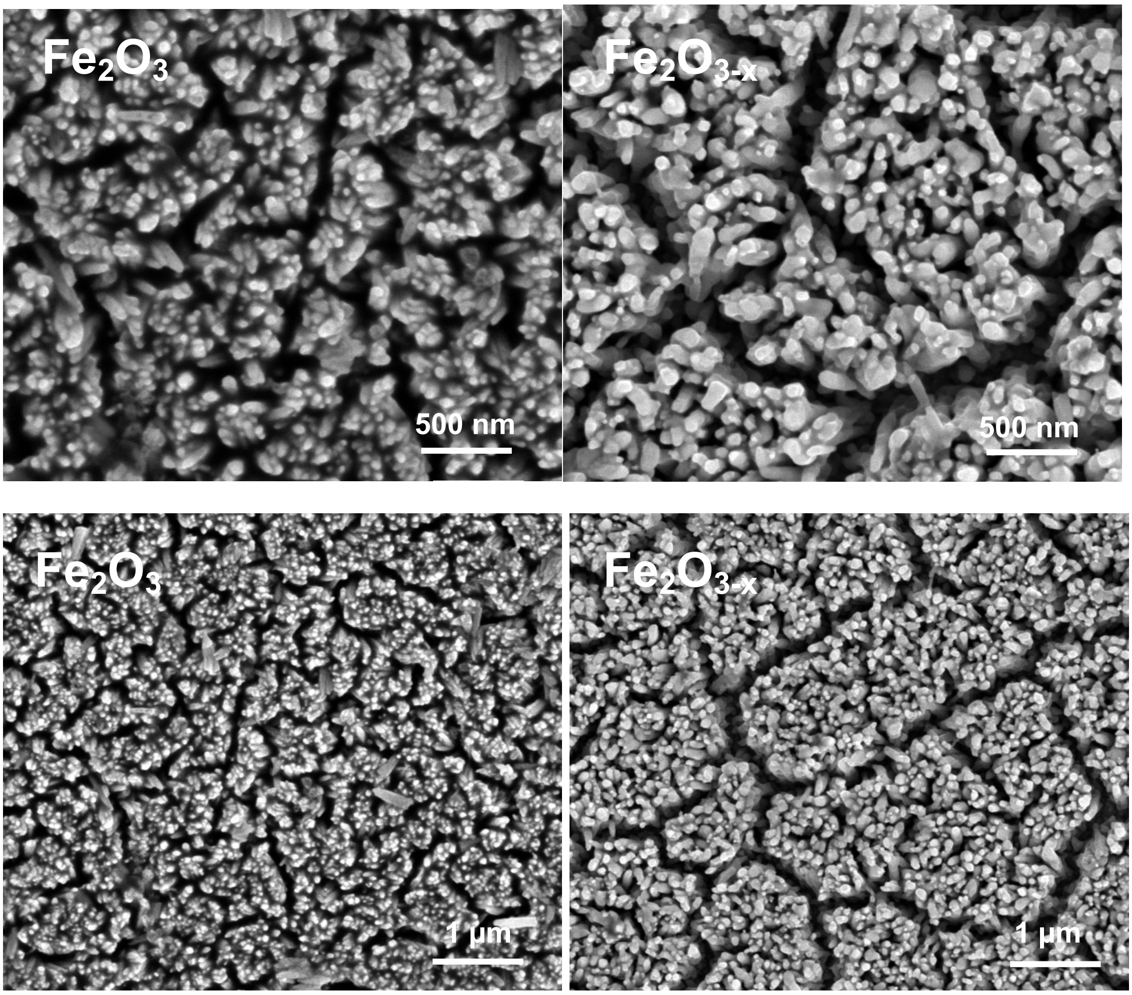

Figure S3. Scanning electron microscopy images of Fe_2O_3 and Fe_2O_{3-x}.

2.4 Scheme of TA assignments at λ > 600 nm and 580 nm

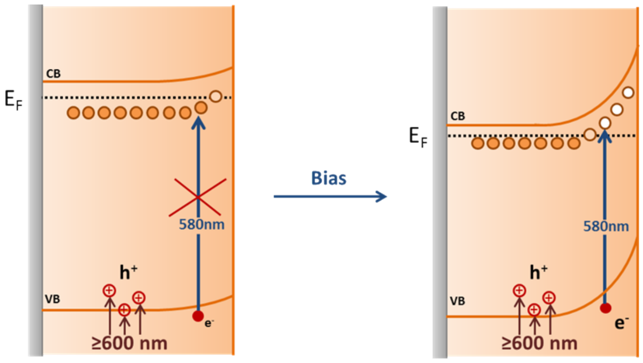

Figure S4. Schematic showing TA assignment in α-Fe_2O_3 of feature at λ > 600 nm to photoholes and bleach at ~580 nm to photo-electron trapping at localized states which is primarily occurring on the sub-microsecond timescale, in line with Barosso *et al.*[4]

Schematic showing the transitions responsible for the broad positive transient absorption measured at wavelengths greater than 600 nm in all of the spectra the and bleach (decrease in optical density) centered at *ca.* 580 nm which is seen to increase in magnitude with applied bias. It has been proposed that the feature at this wavelength is due to localized states close to the band edge, possibly related to the presence of oxygen vacancies.[4,5]

2.5 Steady state photocurrents measurements at various applied potentials

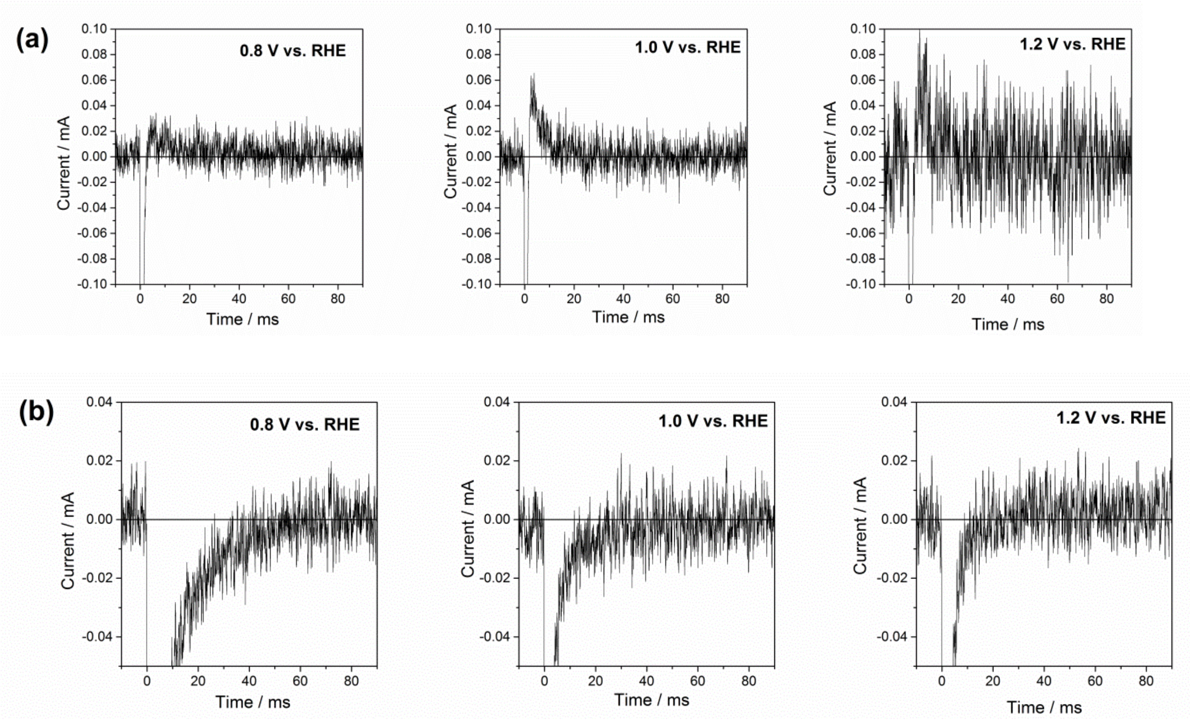

Figure S5. Transient photocurrents measurements at various applied potentials for a) Fe_2O_{3-x} showing no charge re-injected from external circuit and b) Fe_2O_3 showing positive signal on slower timescales where charge is re-injected due to bulk electron-hole recombination on the ms timescale.

2.6 *Overlay of the decay of the TA with the integrated TPC in Fe_2O_3 at 1.4 V_{RHE}*

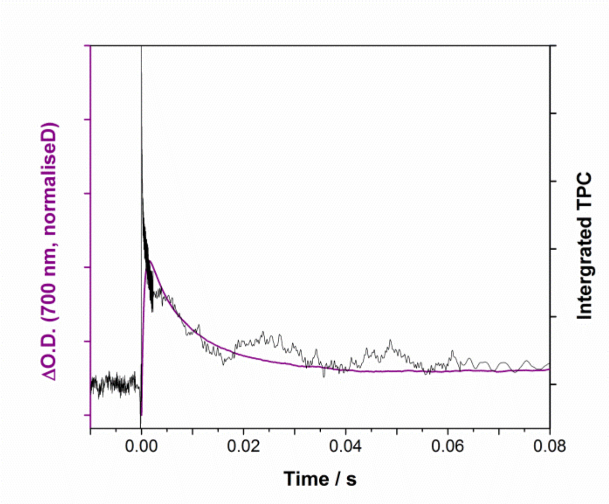

Figure S6. *Overlay of the decay of the TA with the integrated TPC in Fe_2O_3 at 1.4 V (vs. RHE) in 1M NaOH with the photoanodes excited from the electrolyte/electrode side with a UV laser pulse (355 nm, 0.33 Hz).*

An overlay of the kinetics of charge reinjection (TPC) and hole decay (TA) on α-Fe_2O_3 (τ = 10 ms at 1.4 V_{RHE}) shows an excellent agreement indicating that bulk electrons are recombining with the accumulated photoholes measured in the TA experiment, leading to reinjection of electrons from the external circuit.

2.7 Mott-Schottky and space charge layer width calculations

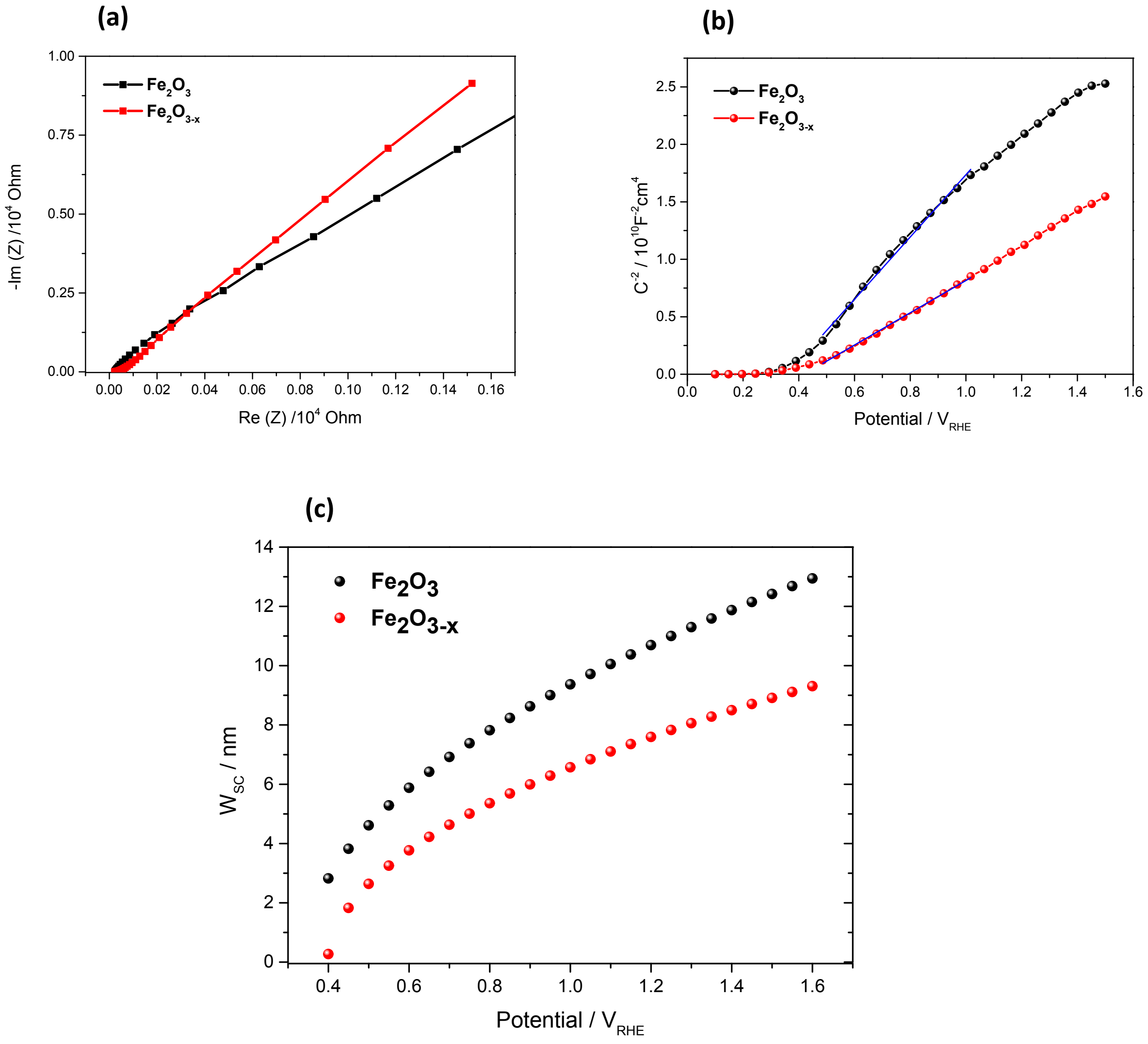

Figure S7. (a) Nyquist impedance plots and (b) Mott-Schottky plots of *α-Fe_2O_3 giving N_d = 6.4 x 10^{19} cm^{-} and E_{FB}= 0.34V and α-Fe_2O_{3-x} giving N_d = 1.2 x 10^{20} cm^{-3} and E_{FB} = 0.40 V.* (c) Space charge layer width calculations.

Great care should be taken when interpreting Mott-Schottky data from highly nanostructured films such as those under study here. Nonetheless the relative change in donor density and the measured flat band potentials are of significance. To provide an indication of how the level of V_o modifies the width of the space charge layer when under a positive bias, and hence the strength of the electric field close to the SCLJ we have calculated representative values of space charge layer width, W_{SC}, for films held at different potentials anodic of flat band (Fig S7c) using the following equation with E being the applied potential:[6]

$$W_{SC} = \sqrt{\frac{2\varepsilon\varepsilon_0}{qN_D}(E - E_{FB})}$$

With the donor densities and flatband potentials obtained to be (α-Fe_2O_{3-x} N_d = 1.2 x 10^{20} cm^{-3}, E_{FB} = 0.40 V and α-Fe_2O_3 N_d = 6.4 x 10^{19} cm^{-3}, E_{FB} = 0.34 V).[7]

These calculations show that the large increase in N_d in the α-Fe_2O_{3-x} electrode significantly decreases the width of the space charge layer (W_{sc}) which is calculated to go from 12 nm for α-Fe_2O_3 to 8 nm for α-Fe_2O_{3-x} at 1.4 V. Due to the limited stability of the Al_2O_3 layer in the electrolyte Mott-Schottky analyses were not completed for the ALD coated samples.

From the absorption spectrum at 355 nm and the known pump light intensity of *ca.* 100 μJ cm^{-2} we can estimate the photo-generated carrier density and compare it to the N_d. We calculate the number of incoming photons to be on the order of $1.78x10^{14}$ cm^{-2} demonstrating that expected oxygen vacancy concentration (assumed to be the difference in N_d) is much greater than the photo-generated charge carrier density.

2.8 Linear sweep voltammograms in the presence of H_2O_2 hole scavenger

In the presence of 0.5 MH_2O_2 we observe a photocurrent at potentials as low as 0.7 VRHE on both α-Fe_2O_3 and α-Fe_2O_{3-x} confirming that (i) initial charge separation is occurring to some degree in both materials at potentials well below the water oxidation photocurrent onset potential and (ii) that in both samples the photoholes are able to reach the surface to participate in oxidation reactions. The significantly higher photocurrent for the oxygen deficient sample in the presence of H_2O_2 (*ca.* x2) is in line with expectations that the greater degree of band bending in Fe_2O_{3-x} enables higher initial charge separation efficiencies. It is significant to note however that although this measurement indicates that the initial charge separation efficiency in Fe_2O_{3-x} is approximately x2 that of Fe_2O_3 this is intself is not sufficient to explain the complete lack of water splitting activity on Fe_2O_3. This is in good agreement with Figure 3 of the main text which also shows that a significant population of photoholes remain in Fe_2O_3 on the micro- to millisecond timescale in NaOH at 1.4 $V_{RHE.}$

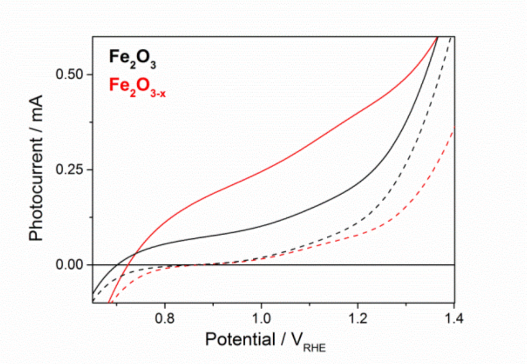

Figure S8. *Linear sweep voltammograms for α-Fe_2O_3 (black) and α-Fe_2O_{3-x} (red), 5 mVs^{-1}, under white light illumination using a 75 W Xe lamp (solid line, ca.10 mW cm^{-2}) and in the dark (dashed line) in 1M NaOH with the presence of a hole scavenger (0.5 M H_2O_2).*

2.9 *Fitting parameters for 700 nm TA signals*

Figure 6a was fitted to the flowing equation:

$$\Delta OD\,(t) = A_1 e^{-(t/\tau_1)^{\beta_1}} + A_2 e^{-(t/\tau_2)^{\beta_2}} + A_3 e^{-(t/\tau_3)^{\beta_3}} + y_0$$

Magnitude	*Lifetime (s)*	*Stretching factor*
**A_1: 2.15E^{-4}**	τ_1: 5.17E^{-4}	β_1: 0.30
**A_2: 5.78E^{-5}**	τ_2: 0.025	β_2: 0.54
**A_3: 6.06E^{-5}**	τ_3: 0.28	β_3: 0.69

2.10 Plot of photocurrent vs. 700 nm hole intensity

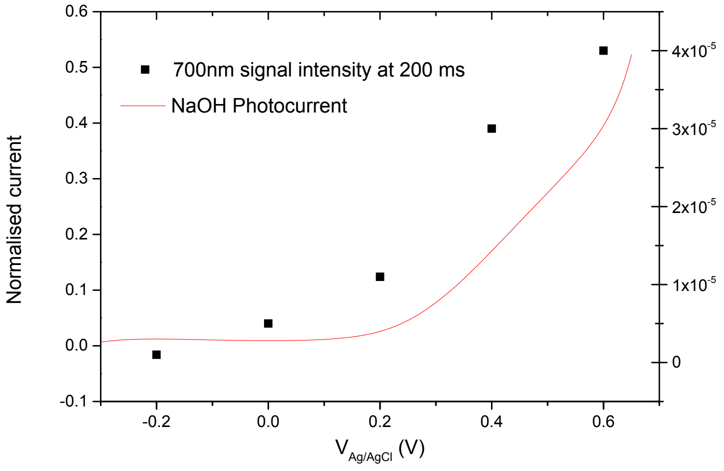

Figure S9. *A plot of the yield of very long-lived photoholes (at 200 ms) versus the applied bias which strongly correlates with the measured photocurrent response of α-Fe_2O_{3-x}.*

2.11 Photocurrents for Al_2O_3 coated Fe_2O_3

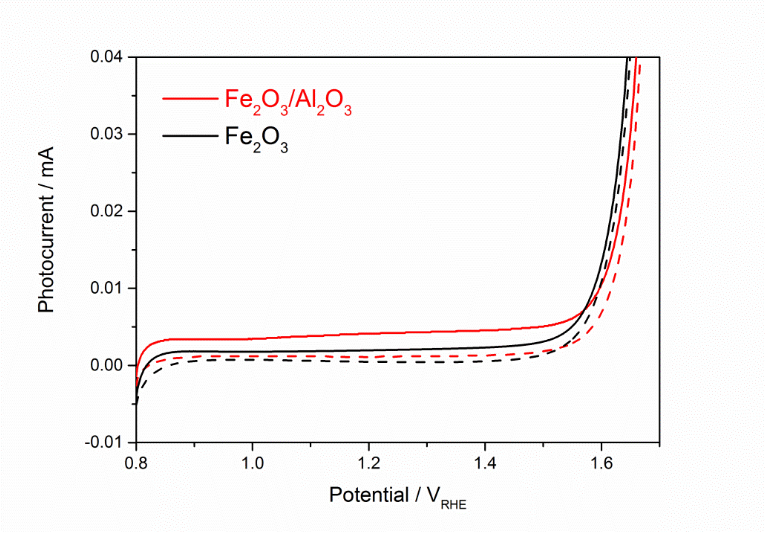

Figure S10. *Photocurrent of α-Fe_2O_3 before (black) and after deposition of ALD Al_2O_3 layer (1 nm, red trace) recorded using a 75 W Xe lamp, dark traces are shown as dashed lines.*

Unlike in $Fe_2O_{3\text{-}x}$, the addition of an Al_2O_3 ALD layer onto an air annealed α-Fe_2O_3 sample showed no improvement in activity confirming that sub-surface oxygen vacancies are required for photoelectrochemical activity in these otherwise un-doped samples.

2.12 Photohole (700 nm) intensities before and after Al_2O_3 coating

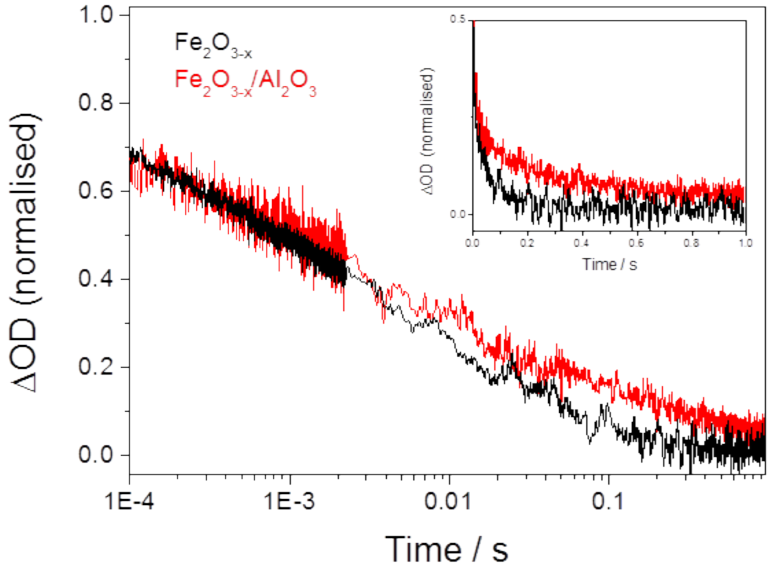

Figure S11. *TA traces recorded at (a) 700 nm (photoholes) in α-$Fe_2O_{3\text{-}x}$ (black) and α-$Fe_2O_{3\text{-}x}/Al_2O_3$ at 1.4 V_{RHE} following UV excitation (355 nm) in 1 M NaOH.*

2.13 Fitting of TA trace recorded at (a) 700 nm (photoholes) in α-Fe2O3-x in the presence of hydrogen peroxide hole scavenger

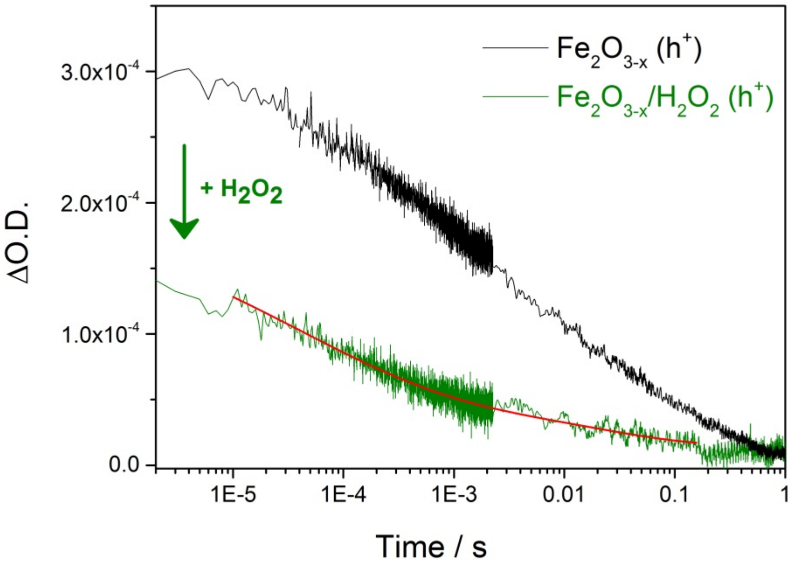

Figure S12. Fitting of ***TA trace recorded at (a) 700 nm (photoholes) in α-Fe_2O_{3-x} in the presence of hydrogen peroxide hole scavenger at 1.4 V_{RHE} following UV excitation (355 nm) in 1 M NaOH (red line on green curve).***

The TA trace measured at 700 nm (holes) in the presence of hydrogen peroxide hole scavenger was found to be well fitted to a bi-exponential decay of the form.

$$\Delta OD\,(t) = A_1 e^{-(t/\tau_1)^{\beta_1}} + A_2 e^{-(t/\tau_2)^{\beta_2}} + y_0$$

Magnitude	*Lifetime (s)*	*Stretching factor*
A_1: 1.14E^{-4}	τ_1: 3.5 x 10^{-5}	β_1: 0.30
A_2: 2.73E^{-5}	τ_2: 2.58 x10^{-2}	β_2: 0.54

With A_1 corresponding to the yield of holes undergoing bulk recombination accounting for the vast majority of the signal. A_2, the relative yield of holes undergoing trap mediated recombination is decreased compared to studies in the absence of H_2O_2 (ESI 2.9), indicating a large decrease in surface hole concentration of in the presence of H_2O_2. In contrast to experiments solely in 1 M NaOH no slow hole injection term is identified. This is in agreement with past studies which suggested that scavenging of surface holes by H_2O_2 is an ultrafast process, occurring on timescales faster than studied here (2 μs - 1 s).[8] We are therefore able to assign that the a significant population of holes transfers to the SCLJ on the sub 2 microsecond timescale, in agreement with a recent fs-TAS study.[9] We note that the low

concentration of surface trapped holes remaining here likely to be due to the limited availability of H_2O_2 at the surface proposed to be due the finite concentration of H_2O_2 present at the electrode surface under the conditions examined (500 mM H_2O_2 in the bulk solution).

References

1. F. Le Formal, N. Tétreault, M. Cornuz, T. Moehl, M. Grätzel, and K. Sivula, *Chem. Sci.*, 2011, **2**, 737.
2. A. J. Cowan, J. Tang, W. Leng, J. R. Durrant, and D. R. Klug, *J. Phys. Chem. C*, 2010, **114**, 4208–4214.
3. S. R. Pendlebury, A. J. Cowan, M. Barroso, K. Sivula, J. Ye, M. Grätzel, D. R. Klug, J. Tang, and J. R. Durrant, *Energy Environ. Sci.*, 2012, **5**, 6304.
4. M. Barroso, S. R. Pendlebury, A. J. Cowan, and J. R. Durrant, *Chem. Sci.*, 2013, **4**, 2724.
5. K. Kobayashi, G. Okada, and J. Kumanotani, *J. Mater. Sci. Lett.*, 1988, **7**, 3–4.
6. L. Steier, I. Herraiz-cardona, S. Gimenez, F. Fabregat-santiago, J. Bisquert, S. D. Tilley, and M. Grätzel, *Adv. Funct. Mater.*, 2014, DOI: 10.1002/adfm.201402742.
7. Y. Ling, G. Wang, J. Reddy, C. Wang, J. Z. Zhang, and Y. Li, *Angew. Chem. Int. Ed. Engl.*, 2012, **51**, 4074–9.
8. K. Sivula, *J. Phys. Chem. Lett.*, 2013, **4**, 1624–1633.
9. S. R. Pendlebury, X. Wang, F. Le Formal, M. Cornuz, A. Kafizas, S. D. Tilley, M. Grätzel, and J. R. Durrant, *J. Am. Chem. Soc.*, 2014, **136**, 9854–7.
